# Resilient Health System As Conceptual Framework for Strengthening Public Health Disaster Risk Management: An African Viewpoint

**DOI:** 10.3389/fpubh.2017.00263

**Published:** 2017-09-28

**Authors:** Olushayo Olu

**Affiliations:** ^1^World Health Organization, Kigali, Rwanda

**Keywords:** resilient health system, public health, disaster risk management, conceptual framework, Africa, opinion article

## Introduction

Disasters whether natural or man-made, pose major challenge to human health and development in Africa; their impact on the health of individuals and communities are often severe and could hinder attainment of global, regional, and national development goals (1–3). Recent disasters in Africa aptly illustrate the complex interaction between health systems and disasters; a vicious cycle in which weak health systems provide fertile grounds for deterioration of public health and natural hazards into disasters while on the other hand, disasters further decimate already weak health systems (4). The sustained transmission of the 2014/15 Ebola virus disease outbreak in Guinea, Liberia, and Sierra Leone was consistently linked to the weak health systems in these countries (5, 6). The outbreak resulted in the death of several health workers (7), depletion of scarce financial resources, diversion of medical equipment. This in addition to overburdening of already weak health information and supply chain management systems resulted in disruption of health services delivery in these countries (8–10). Other disasters such as the Yellow Fever outbreaks in Angola, Democratic Republic of Congo and Uganda, and ongoing armed conflicts in South Sudan, Central Africa Republic, northeast Nigeria, and other African countries also had similar consequences (11–15). This pattern is not limited to Africa; the fragile pre-disaster health systems in the city of New Orleans in America and the Eastern Visayas Region of the Philippines contributed to the public health consequences of Hurricane Katrina and Haiyan (Yolanda) and constrained timely and effective post-disaster health system recovery efforts (16, 17). The pre-Katrina health system in the city of New Orleans was characterized by low coverage of health insurance and reduced access to health services by the largely poor population of the city (16). Similar challenges such as inadequate health-care infrastructure, staffing, and low coverage of health insurance, which reduced access to health services were also prevalent in the affected areas of the Philippines pre-Hurricane Haiyan (17).

The Sendai Framework for Disaster Risk Reduction (SFDRR) and sustainable development goals (SDGs), both of which are landmark United Nations agreements adopted in 2015, recommend scaling up implementation of disaster risk reduction (DRR) strategies as means to improve resilience to disasters globally (18, 19). The SFDRR in contrast to its predecessor, the Hyogo Framework for Action, puts a lot of emphasis on health (20). It proposes resilient health systems as an opportunity for ensuring effective DRR in the health sector (20). The World Health Assembly, through resolution 64.10, urged countries to strengthen disaster risk management (DRM) programs by incorporating them into national health systems (21). The 2008 Ouagadougou declaration on Primary Health Care and the African Regional strategy for DRM in the health sector also advocated for the use of strong health systems as the basis for addressing the health vulnerabilities and inequalities, which are associated with disasters in Africa (22, 23).

The foregoing and available literatures (2, 24) make a strong case for the use of resilient health systems as a conceptual framework for public health DRM in Africa. Calls for the use of resilient health system as the basis for public health DRM have intensified lately (25); however, there is paucity of practical guidance, requisite tools, and skills for integration of DRM into longer-term health system programs in public health settings in Africa (26). This often results in parallel implementation of health systems strengthening and public health DRM programs within Ministries of Health and between their Disaster Management counterparts with duplication of efforts and lack of synergy. This article reflects on the nexus between the health system framework and DRM and provides insights into how a resilient health system could be used as a framework to strengthen public health DRM in Africa.

## The Health System Framework and DRM

**Figure 1:**
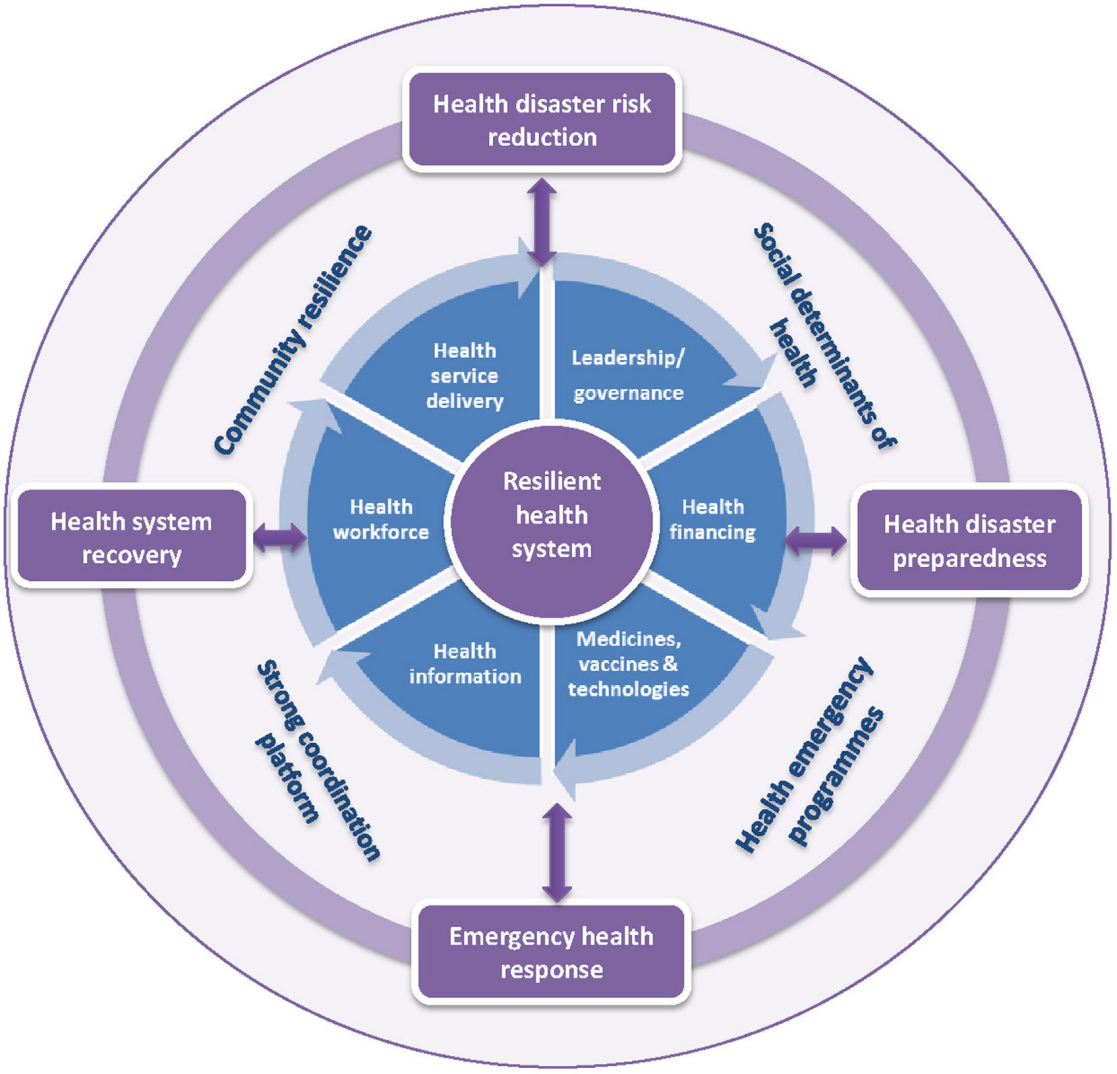
The health system building blocks as a conceptual framework for public health disaster risk management.

The health system consist of “*all organizations, people and actions whose primary intent is to promote, restore, or maintain health*” (27), while DRM is defined as the use of administrative and operational procedures to implement interventions aimed at reducing the adverse impact of disaster hazards (28). The health system encompasses all direct health-improving activities implemented either at home, in the community, and the formal health sector level and the social determinants of health, which are the conditions under which people are born, live, and grow. The health system framework comprises six building blocks namely health service delivery, health workforce, health information management system, medical products including vaccines and technologies, health financing, and health leadership and governance (Figure 1).

A resilient health system is one which is able to effectively prepare for, withstand the stress of, and respond to the public health consequences of disasters (29). Resilient health systems are able to protect themselves and human lives from the public health impact of disasters and are critical to achieving good health outcomes before, during, and after disasters (26). Kruk et al. defined five elements of resilient health systems (29). Resilient health systems should be aware of the strengths and vulnerability of its building blocks and the spectrum of hazards and risks to which it is exposed. They should be able to respond to a wide range of public health issues before or during a disaster. Health systems should be able to quickly and effectively adapt to changing situations and should use integrated approaches for responding to public health events such as disasters. Last, a resilient health system should be able to regulate itself. These elements provide a good basis for strengthening and using health system for public health DRM.

## Resilient Health Systems, Communities, and Social Determinants of Health as Basis for Public Health DRM

Lessons from the West African Ebola outbreak and Hurricanes Katrina and Haiyan show how an emergency situation can deteriorate into a disaster in the face of a weak health system (4, 16, 17). Conversely, resilient health systems could reduce vulnerability to the public health consequences of disasters (4). In the aftermath of a disaster, strong supply chain systems for essential medicines, safe health facilities, and adequate numbers of well-trained health workers would ensure the provision of uninterrupted basic health-care services to disaster affected populations. Functional health information management systems would provide the information required for timely detection and response to presence of biological hazards such as cholera, typhoid fever, watery diarrhea, measles, etc., which often occurs as aftermaths of disasters. Adequate financing of emergency health service programs and strong health governance and oversight systems would ensure that human, financial, and logistics resources are available and utilized to implement well-coordinated DRM strategies to mitigate the public health consequences of the disaster. Good service delivery and coverage of key public health interventions such as immunization, insecticide-treated bed nets, clean water, and improved sanitation would prevent disease outbreaks among disaster-affected populations. These would contribute to good public health outcomes during a disaster.

In practical terms, effective measures to address the public health consequences of droughts such as good immunization coverage, adequate nutrition, and health services delivery including clinical management of severe acute malnutrition, ongoing surveillance of nutrition indicators, and effective risk communication about malnutrition would ensure that such situations do not deteriorate into famines (24). Similarly, safe and well-sited health facilities, good health sector disaster mitigation, contingency and business continuity planning, adequate essential medicines, and supplies for trauma care, and well-trained health staff would ensure that the consequences of earthquakes do not result in major public health disasters (24).

Practical application of resilient health systems as a framework for strengthening public health DRM is, therefore, an imperative in Africa. This requires the strengthening and use of the six health system building blocks as elements in the implementation of public health DRR, preparedness, response and post-disaster recovery interventions at the individual, community, and formal health sector levels (Table 1). Apart from weak health systems, poor status of the social determinants such as poverty, lack of good housing, inadequate access to good nutrition, clean water, improved sanitation, education, and social protection could reduce individual and community resilience and increase the risk of disasters (Figure 1) (2, 30). Thus optimal social determinants of health and resilient communities are also required for mitigating the public health risks and impacts of disasters (2, 31).

**Table 1:** Application of the health system framework to public health disaster risk management (DRM).

Health system building blocks	DRM elements and public health interventions			
	Disaster risk reduction (DRR)	Disaster preparedness	Disaster response	Post-disaster recovery
Health leadership and governance	Development of institutional framework for public health DRR Inclusion of DRR in existing national health policies and strategies Establishment of public health DRR coordination committees Designation of DRR units in MOHs	Development of institutional framework for public health disaster preparedness Health emergency contingency planning Simulation exercises Development of business continuity plans Establishment of public health emergency coordination mechanisms	Development of public health disaster response plans with inputs from all health programs and other relevant sectors Establishment of emergency public health coordination committees Supervision, monitoring, and evaluation of emergency health response	Establishment of coordination structures for implementing health system recovery programs Revise/update hazard specific contingency plans Strengthening regulatory functions of government Facilitation of the review/update of strategies/guidelines of various health programs
Health financing	Development of framework for universal health coverage during disasters Allocation of funds to health DRR	Allocation of funds to public health disaster preparedness Establishment of public health emergency funds	Allocation of funds for public health disaster response Implementation of framework for universal health coverage including implementation of financial risk protection measures and health insurance	Resource mobilization and allocation of funds for health system recovery Allocation of funds for establishment of sustainable health financing systems such as such as community and social insurance Strengthen government financial management systems and establish mechanisms for financial coordination and accountability
Medical products, vaccines, and technologies	Assessment of risks to stockpile of medical products, equipment, and vaccines as part of health Vulnerability and Risk Assessment and Mapping (VRAM) Appropriate siting and storage of medical products, vaccines, and medical equipment	Development of list of essential medicines, emergency health kits, etc. Procurement and prepositioning of emergency health kits Establishment of quality assurance system for essential medicines, kits, etc. Establishment of supply chain systems for medicines, vaccines, and medical equipment Development and implementation of medicines and equipment donation policy	Procurement and deployment of emergency health kits, personal protective equipment, and medical supplies Strengthening of supply chain system for essential medicines, emergency health kits, personal protective equipment, etc.	Strengthen supply chain management system Institution of quality assurance mechanisms for medical products, vaccines, and equipment Development of essential medicines list and guidelines for rational use of medicines and training of health workers Standardization of medical equipment according to levels of care and strengthening maintenance functions and skills Strengthen the cold chain
Health information management	Public health disaster VRAM assessments Implementation of Health Facility Safety Index (HIS) surveys	Establishment of public health early warning systems Establishment of ongoing public health surveillance system (e.g., for diseases and nutrition)	Rapid health assessments Establishment of active disease surveillance system for public health events Health services availability mapping Specialized surveys such as mortality survey, nutrition survey Community surveillance	Post-disaster health needs assessments Strengthen routine disease surveillance and health information management system Health DRM capacity assessments Health services availability mapping
Human resources for health (HRH)	Conduction of training needs assessment for HRH Training of health workers on health DRR Training of HRH on infection prevention and control	Development of terms of reference for HRH Identification and training of rapid health response teams Establishment of roster of emergency public health experts	Re-deployment of existing HRH Recruitment and deployment of additional HRH Establishment of system for protection of HRH (infection prevention and control)	Assess impact of disaster on HRH Develop HRH emergency plans for scaling up capacity for new and/or increased health demands Engage and scale up capacity for the required community health workers in delivery of health services. Institute staff recruitment, training and retention packages Strengthen health training institutions to rapidly increase HRH pool Establish task shifting system among the staff
Service delivery	Retrofitting of at-risk health facilities Review of health facility building codes Use of risk management information to guide siting of public health infrastructure Public health awareness campaigns and community mitigation activities	Public health risk communication Evacuations and preparation of camps, treatment, and isolation centers or shelters	Mass casualty management including medical evacuation Building of temporary public health facilities Public health risk communication Provision of primary health-care services Public preventive services such as immunization, bed nets, etc. Provision of specialized health services such as mental health, HIV/TB, NCD treatment Support to water and sanitation in health facilities Water quality surveillance Infection prevention and control	Define/revise basic health-care package to address existing post-disaster/conflict situation Address equity issues such as religious, ethnic, gender, age, and other socioeconomic factors that negatively influence use of services Develop and disseminate behavioral change communication strategies to improve access. Implement community-based initiatives to improve service coverage

## Conclusion

The foregoing points to weak health system as a key factor, which determines public health disaster risk in Africa and similar settings. Public health DRM programs on the continent should, therefore, place resilient health systems at their core. This calls for use of innovative solutions, which are adapted to the African context to build the resilience of health systems and communities. This could be achieved through the use of the five elements proposed by Kruk et al. namely awareness, diversity, self-regulation, integration, and adaptability (29). In addition, the social determinants would also need to be strengthened as basis for reinforcing public health DRM in Africa. Furthermore, African health systems should be protected from the adverse impact of disasters in order to preserve the gains made during the millennium development goal era and contribute to the attainment of global and regional development goals such as the SDG and Africa’s Agenda 2063. These requires a number of actions.

First, African countries should regularly conduct independent assessments of the resilience of their health systems vis-à-vis their capacity for DRM by conducting health vulnerability and risk assessments as part of joint external evaluation of the International Health Regulations core capacities. Second, the countries should develop and implement practical policies, strategies, and guidelines to strengthen health system and community resilience. Integration of DRM strategies into long-term health systems development programs should also be institutionalized. Similarly, health system strengthening strategies should be mainstreamed into public health DRM programs. Third, individual and institutional capacity building to improve the skills and knowledge base for health system resilience building and public health DRM should be scaled up on the continent. Furthermore, guidelines and tools for practical application of resilient health system building blocks as elements in the implementation of public health DRM programs are required. Fourth, the use of multisectoral and multi-disciplinary approach to ensure that the issues of community resilience and social determinants are jointly addressed as part of holistic public health DRM programs is also an imperative. Fifth, operational researches to identify novel mechanisms for applying the health system framework to public health DRM should be intensified. Such researches should also examine the disparities between the health systems in developed and developing countries and whether these translate to real differences in public health outcomes during disasters. Importantly, these actions should be implemented as comprehensive packages, which are integrated into SFDRR and SDGs domestication programs and national health sector policies and strategic plans to ensure coherence, synergy, and sustainability.

## Author Contributions

OO is the corresponding author of this manuscript. He solely designed and wrote the work described in it.

## Conflict of Interest Statement

The author declares that this manuscript was written in the absence of any commercial or financial relationships that could be construed as a potential conflict of interest.

## Abbreviations

DRM, disaster risk management; SDGs, sustainable development goals; SFDRR, Sendai Framework for Disaster Risk Reduction.

## References

[1] Michailof S Kostner M Devictor X Post-conflict recovery in Africa: an agenda for the African region. Africa Region Working Paper Series No 30. World Bank. (2002). Available from: http://www.sergemichailof.fr/wp-content/uploads/2010/02/postconflictrecoveryinafrica2002.pdf

[2] Dar O Buckley EJ Rokadiya S Huda Q Abrahams J. Integrating health into disaster risk reduction strategies: key considerations for success. Am J Public Health (2014) 104(10):1811–6. 10.2105/AJPH.2014.302134 25122022 PMC4167116

[3] Lamptey BJ Awojobi ON The spread of the Ebola virus disease and its implications in the West African sub-region. Int J Innov Sci Res (2014) 11(1):130–43.

[4] Kieny MP Evans DB Schmets G Kadandale S Health-system resilience: reflections on the Ebola crisis in western Africa. Bull World Health Organ (2014) 92(12):850 10.2471/BLT.14.149278 25552765 PMC4264399

[5] Kieny MP Dovlo D Beyond Ebola: a new agenda for resilient health systems. Lancet (2015) 385(9963):91–2. 10.1016/S0140-6736(14)62479-X 25706456 PMC5513100

[6] Cancedda C Davis SM Dierberg KL Lascher J Kelly JD Barrie MB Strengthening health systems while responding to a health crisis: lessons learned by a nongovernmental organization during the Ebola virus disease epidemic in Sierra Leone. J Infect Dis (2016) 214(Suppl 3):S153–63. 10.1093/infdis/jiw345 27688219 PMC5050485

[7] Health Worker Ebola Infections in Guinea. Liberia and Sierra Leone – A Preliminary Report. World Health Organization (2015). Available from: http://apps.who.int/iris/bitstream/10665/171823/1/WHO_EVD_SDS_REPORT_2015.1_eng.pdf?ua=1&ua=1

[8] Brolin Ribacke KJ Saulnier DD Eriksson A von Schreeb J Effects of the West Africa Ebola virus disease on health-care utilization – a systematic review. Front Public Health (2016) 4:222 10.3389/fpubh.2016.00222 27777926 PMC5056406

[9] Elston JW Cartwright C Ndumbi P Wright J. The health impact of the 2014–15 Ebola outbreak. Public Health (2017) 143:60–70. 10.1016/j.puhe.2016.10.020 28159028

[10] Bolkan HA Bash-Taqi DA Samai M Gerdin M von Schreeb J. Ebola and indirect effects on health service function in Sierra Leone. PLoS Curr (2014) 6. 10.1371/currents.outbreaks.0307d588df619f9c9447f8ead5b72b2d 25685617 PMC4318968

[11] Green A Yellow fever continues to spread in Angola. Lancet (2016) 387(10037):2493 10.1016/S0140-6736(16)30835-2 27353673

[12] Nishino K Yactayo S Garcia E Aramburu GJ Manuel E Costa A Yellow fever urban outbreak in Angola and the risk of extension. Wkly Epidemiol Rec (2016) 91(14):186–92. 27066610

[13] Jones A Howard N Legido-Quigley H. Feasibility of health systems strengthening in South Sudan: a qualitative study of international practitioner perspectives. BMJ Open (2015) 5:e009296. 10.1136/bmjopen-2015-009296 26700280 PMC4691708

[14] Central African Republic Crisis and Its Regional Humanitarian Impact. United Nations Office for Coordination of Humanitarian Affairs (UNOCHA). (2014). Available from: https://www.humanitarianresponse.info/system/files/documents/files/Central%20African%20Republic%20Crisis%20and%20its%20Regional%20Humanitarian%20Impact%20June%202014.pdf

[15] Omole O Welye H Abimbola S Boko Haram insurgency: implications for public health. Lancet (2015) 385(9972):941 10.1016/S0140-6736(15)60207-0 25747581

[16] Rudowitz R Rowland D Shartzer A. Health care in New Orleans before and after hurricane Katrina. Health Aff (2006) 25(5):w393–406. 10.1377/hlthaff.25.w393 16940307

[17] Casamina C Lee C Reyes R Tropical cyclone Haiyan/Yolanda medical relief mission: perspectives of John A Bums School of Medicine 2^nd^ year medical students. Hawaii J Med Public Health (2015) 74(5):176–8. 26019988 PMC4443618

[18] Sendai Framework for Disaster Risk Reduction 2015–2030. (2015). Available from: http://www.unisdr.org/we/coordinate/sendai-framework

[19] Sustainable Development Goal 3: Ensure Healthy Lives and Promote Well-Being for All at All Ages. (2016). Available from: http://www.un.org/sustainabledevelopment/health/

[20] Aitsi-Selmi A Murray V. Protecting the health and well-being of populations from disasters: health and health care in the Sendai framework for disaster risk reduction 2015–2030. Prehosp Disaster Med (2016) 31(1):74–8. 10.1017/S1049023X15005531 26675042

[21] World Health Assembly Resolution. (2011). Available from: http://apps.who.int/iris/handle/10665/3566

[22] Disaster Risk Management: A Strategy for the Health Sector in the African Region. Report of the Secretariat. World Health Organization, Regional Committee for Africa. Luanda, Republic of Angola: World Health Organization (WHO) (2012). Available from: http://apps.who.int/iris/bitstream/10665/80238/1/AFR-RC62-6-e.pdf

[23] Barry SP Somanje H Kirigia JM Nyoni J Bessaoud K Trapsida JM The Ouagadougou declaration on primary health care and health systems in Africa: achieving better health for Africa in the new millennium. African Health Monit (2010) (12). Available from: https://www.aho.afro.who.int/en/ahm/issue/12/reports/ouagadougou-declaration-primary-health-care-and-health-systems-africa-achieving

[24] Bayntun C Rockenschaub G Murray V. Developing a health system approach to disaster management: a qualitative analysis of the core literature to complement the WHO Toolkit for assessing health-system capacity for crisis management. PLoS Curr (2012) 4:e5028b6037259a. 10.1371/5028b6037259a 23066520 PMC3461970

[25] Aitsi-Selmi A Murray V The Sendai framework: disaster risk reduction through a health lens. Bull World Health Organ (2015) 93(6):362 10.2471/BLT.15.157362 26240454 PMC4450716

[26] Bayntun C. A health system approach to all-hazards disaster management: a systematic review. PLoS Curr (2012) 4:e50081cad5861d. 10.1371/50081cad5861d 23066519 PMC3461969

[27] Everybody’s Business. Strengthening Health Systems to Improve Health Outcomes; WHO’s Framework for Action. World Health Organization (WHO) (2007). Available from: http://www.who.int/healthsystems/strategy/everybodys_business.pdf

[28] UNISDR Terminology on Disaster Reduction. United Nations Office for Disaster Risk Reduction (UNISDR). (2009). Available from: http://www.unisdr.org/files/7817_UNISDRTerminologyEnglish.pdf

[29] Kruk ME Myers M Varpilah ST Dahn BT What is a resilient health system? Lessons from Ebola. Lancet (2015) 385(9980):1910–2. 10.1016/S0140-6736(15)60755-3 25987159

[30] Acharya M Ebola virus disease outbreak – 2014: implications and pitfalls. Front Public Health (2014) 2:263 10.3389/fpubh.2014.00263 25520949 PMC4249494

[31] Marmot M Friel S Bell R Houweling TA Taylor S Commission on Social Determinants of Health Closing the gap in a generation: health equity through action on the social determinants of health. Lancet (2008) 372(9650):1661–9. 10.1016/S0140-6736(08)61690-6 18994664

